# New therapeutic strategies to treat human cancers expressing mutant p53 proteins

**DOI:** 10.1186/s13046-018-0705-7

**Published:** 2018-02-15

**Authors:** Giovanni Blandino, Silvia Di Agostino

**Affiliations:** Oncogenomic and Epigenetic Unit, Department of Diagnostic Research and Technological Innovation, IRCCS Regina Elena National Cancer Institute, 00144 Rome, Italy

## Abstract

The tumor suppressor p53 plays a critical role to preserve DNA fidelity from diverse insults through the regulation of cell-cycle checkpoints, DNA repair, senescence and apoptosis. The *TP53* is the most frequently inactivated gene in human cancers. This leads to the production of mutant p53 proteins that loose wild-type p53 tumor suppression functions and concomitantly acquire new oncogenic properties among which deregulated cell proliferation, increased chemoresistance, disruption of tissue architecture, promotion of migration, invasion and metastasis and several other pro-oncogenic activities. Mouse models show that the genetic reconstitution of the wild type p53 tumor suppression functions rescues tumor growth. This strongly supports the notion that either restoring wt-p53 activity or inhibiting mutant p53 oncogenic activity could provide an efficient strategy to treat human cancers. In this review we briefly summarize recent advances in the study of small molecules and compounds that subvert oncogenic activities of mutant p53 protein into wt-p53 tumor suppressor functions. We highlight inhibitors of signaling pathways aberrantly modulated by oncogenic mutant p53 proteins as promising therapeutic strategies. Finally, we consider the clinical applications of compounds targeting mutant p53 and the use of currently available drugs in the treatment of tumors expressing mutant p53 proteins.

## Background

In the recent years, several consortia have led the sequencing of human cancer genomes identifying a myriad of genomic and chromosomal alterations in many human cancers [1–10]. Among them, the gene most commonly mutated is *TP53*: 96% in ovarian serous carcinoma [7], 85% in small cell lung cancer [5], 75% in pancreatic cancer [8], 60% in head and neck squamous cell carcinoma [9], 54% in invasive breast carcinoma [10], just to mention few tumor types. Approximately 70% of *TP53* mutations are predominantly missense in one allele with loss of the second allele by loss of heterozygosity (LOH). Most of the missense mutations reside in the p53 DNA-binding region and can be classified as either contact (interfere directly with DNA binding) or conformational (induce local or global conformational distortions) mutations [11, 12]. Several ‘hotspot’ mutations can be distinguished, such as R175, G245, R248, R249, R273 and R282, which represent about 30% of all mutations in *TP53* across all human cancer types [13].

In several human tumours specific *TP53* mutations have been associated to poor prognosis [14, 15]. In line with this, in patients affected by the Li-Fraumeni (LF) syndrome, germline missense p53 mutations have been associated with earlier age of tumour onset when compared to germline *TP53* loss [16]. The tumoral and metastatic phenotype of mutant TP53-bearing tumours can be also ascribed to specific mutant p53 protein-protein interactions. Indeed, it has shown that mutp53 can drive the expression of key regulators of proliferation, invasion and metastasis through the binding to several transcription factors including NF-Y, E2Fs, NFkBp65, NfkBp50, SREBP, YAP, VDR or NRF2. This leads to increased proliferation, cholesterol synthesis, inhibition of autophagy and DNA repair machinery, accumulation of reactive oxygen species, and enhanced cell survival [17–26]. In this scenario mutant p53 functions as a co-factor able to sustain the expression of several pro-oncogenic genes that affect diverse signalling pathways whose aberrant activation contributes to increased proliferation, enhanced metastatic potential and acquisition of resistance to specific therapies.

Induction of cell migration by mutp53 is highly cell-context-dependent, and additional signals such as oncogenic Ras in combination with receptors including transforming growth factor β (TGFβ) receptor, epidermal growth factor receptor (EGFR), and MET might be required to support this activity [27–30]. Mechanistically, TGFβ acts in concert with oncogenic Ras and mutp53 to induce the assembly of a mutp53/p63 protein complex in which SMADS serve as platform to sequester and inhibit metastasis suppressor p63 target genes [27]. By inhibiting p63, mutp53 can regulate a pro-invasive transcription program that includes regulation of the expression of Dicer, DEPDC1, Cyclin G2, and SHARP1 [27, 31]. The interaction of mutp53 with p63 also enhances the recycling and signalling of cell surface receptors, by engaging the RAB11 effector, RAB coupling protein (RCP). Indeed, expression of mutp53, or inhibition of p63, promotes RCP-mediated recycling of growth factor receptors such as the EGFR and MET [28–30]. Consistently, the binding of mutp53 to p73 inhibits the triggering of apoptosis p73-dependent in response to chemotherapeutic treatment [32, 33], an activity that requires TopBP1 to prevent binding of p73 to target gene promoters [34]. Although mutant p53 likely functions by modulating p63 and p73 activity, further studies are required to clarify effects on different isoforms and different outcomes.

Mutations of *TP53* are typically seen in the later clinical stages of cancers and the genomic instability is one of the most important features of tumors expressing mutant p53 proteins [35]. According with the oncogene-induced DNA damage model, activated oncogenes induce in both precancerous lesions and established cancers an aberrant DNA damage response (DDR), the failure of DNA replication forks regulation and the formation of DNA double strand breaks (DSBs) [6, 35–37]. This continuous accumulation of DNA alterations activates *TP53,* which exerts its safeguard mechanism by promoting apoptosis or senescence [6, 35–37]. When this replicative stress contributes to increase the selective pressure to mutate *TP53,* the DDR is definitively compromised allowing cancers to develop and to spread [6, 35–37]. The high prevalence and tumor specificity render mutant p53 proteins very attractive targets for cancer therapy. As mutant p53 is still rather difficult to tackle efficaciously, the identification of mutant p53 tumor dependencies might alternative targeting opportunities.

### Mutant p53 proteins in human cancers

Among human cancers with *TP53* missense mutations, about 60% show concomitant deletion of the other allele [38]. The remaining group (40%) does not undergo LOH, retaining a wild-type TP53 allele. In cancer cells that do not undergo LOH of the wild-type TP53 allele, wild-type p53 expression and function may be inhibited through a dominant negative mechanism. Indeed, mutant p53 proteins are able to heterodimerize with wild-type p53 proteins, forming complexes that attenuate the function of wild-type p53 though conformational shifts or inhibiting the DNA-binding activity of wild-type p53 on target genes [39, 40].

Unlike wtp53 protein, mutant p53 is a rather stable protein. Efficient mutp53 GOF activity requires elevated levels of mutp53 protein in the cancer cell. Although transcriptional and translational mechanisms contribute to such increase, its main driving mechanism is believed to be the increased protein stability of mutp53. The majority of the regulatory pathways of p53 are shared between wt-and mutant p53 proteins. However, a number of key differences account for the chronic stabilization and activation of mutant p53, which drive its oncogenic GOF.

In primary cells derived from human Li-Fraumeni syndrome patients, who carry germline p53 mutations, the levels of mutp53 are rather low and comparable to those of wtp53 [41]. This and other works corroborate the hypothesis that mutp53 is not intrinsically stable, rather, alterations that occur within tumor cells lead to its stabilization. Accordingly, mutp53 protein levels are steady-state in mutp53 knockin mice, but significantly increase in tumors that arise in these mice [36, 42]. Subsequent studies have shown that multiple oncogenic effects can stabilize mutant p53 in vivo and drive its oncogenic functions.

The degradation of wtp53 involves several E3 ubiquitin ligases that target p53 for polyubiquitylation and consequent proteasomal degradation, as well as ubiquitin-independent degradation in the proteasome. The most important driver of p53 degradation is the ubiquitin ligase mouse double-minute 2 (MDM2) that targets p53 to the proteasome for degradation [43–45]. MDM2 is itself a p53 target gene, its induction by p53 results in a negative feedback loop through which both proteins are kept to steady state levels [46]. However, mutp53 fails to transactivate the MDM2 gene. MDM2 protein levels are rather low in cells that express only mutp53. Indeed, ablation of endogenous ubiquitin ligases MDM2 and p16INK4a in mutp53 knockin mice leads to a substantial increase in endogenous mutp53 levels [36, 47]. The ubiquitination of mutp53 is also enhanced by the activity of other E3 ligases, CHIP and Cop1 although the overall efficiency of mutp53 ubiquitination is reduced compared with that of wt-p53 protein [45].

Mutation of p53 may affect its stability through a combination of mutant p53 inherent biochemical and biophysical properties as well as pathways aberrantly activated in genetically damaged cells. Hence, in primary cells derived from mutp53 knockin mice mutp53 can be stabilized by genotoxic stress better than wtp53 [42]. Furthermore p53 LOH seems to be a necessary prerequisite for mutant p53 stabilization and gain-of-function in vivo [48]. The heat shock protein HSP90 chaperone machinery is highly activated in cancer versus normal tissues, rendering them resistant to drugs by supporting proper folding of conformationally oncoproteins including mutp53 [49, 50]. Both structural and DNA-contact classes of mutant p53 proteins require HSP90 to escape degradation by their E3 ubiquitin ligases [51]. Interestingly, the inhibition of HSP90 by 17AAG or siRNA against HSF1 (a major transcription factor for HSP) enhances the ubiquitination and degradation of mutp53 [52]. 17AAG and its hydrophilic derivative 17DMAG are ansamycin-derived highly specific first generation HSP90 inhibitors (Hsp90i) [53]. Likewise, histone deacetylase inhibitors (HDACi), including FDA-approved SAHA, are promising anti-cancer drugs whose actions involve hyperacetylation of histone and select non-histone targets including HDAC6 substrate HSP90, thus indirectly inhibiting HSP90 [54].

Further work will reveal additional mechanisms that lead to mutp53 stabilization in cancer cells, thereby sustaining its GOF activities.

### Downstream effector pathways related to mutant p53

At a cellular level mutp53 GOF activity has been shown to abrogate all or most of the cellular responses mediated by wtp53 such as cell-cycle arrest, apoptosis, senescence, DNA repair, maintenance of centrosome number, restriction of phenotypic plasticity, and preservation of homeostatic balance [14, 55, 56]. In addition, the presence of mutant p53 compromises cellular responses to therapeutic agents [57–59], increases inflammation and angiogenesis [19, 59] and correlates with enhanced metastasis and aggressiveness [14, 60, 61].

Accumulating evidence has suggested the involvement of mevalonate pathway in cancer progression. Increased expression of mevalonate pathway-associated proteins is correlated with poor prognosis in breast cancer patients [62, 63]. The mevalonate pathway is an essential lipogenic and uses acetyl-CoA to produce isoprenoids and cholesterol [62]. Isoprenoids are required for protein prenylation/lipidation (farnesylation and geranylgeranylation), which enables target proteins, including Ras and Rho small guanosine triphosphatases (GTPases), to anchor to the cell membrane. Cholesterol is used as an important hydrophobic precursor to bile acids, hormones, and lipoproteins [62].

Growing evidence reported the functional association between the mevalonate pathway and oncogenic proteins including mutp53, Ras, Rho, and YAP/TAZ [21, 64, 65]. Recently, through independent high-throughput screening of chemical compound libraries, statins have been identified as drugs that deplete mutp53 [66] and interfere with YAP/TAZ oncogenic activity in cancer cells [22, 23]. Yes-associated protein (YAP) and its paralogue TAZ are transcriptional coactivators and downstream effectors of the Hippo signaling pathway which regulates organ growth, tissue regeneration, and stemness [67]. Also, the roles of YAP and TAZ in cancer progression are well defined [68]. In breast cancer cells, oncogenic mutant p53 acts as a positive transcriptional cofactor for SREBPs, leading to elevated expression of mevalonate enzymes [21] and SREBP-mevalonate axis is a relevant input for YAP/TAZ oncogenic activity [23]. Statins mainly deplete conformational or misfolded mutp53 with minimal impact on wtp53 and DNA-contact mutp53 that maintain native protein structure. Statin’s effect is specific to the mevalonate pathway, because mutp53 depletion by statins is rescued by supplementation with MVA, a metabolite produced by HMGCoAR enzyme [66]. Although many clinical works support the positive roles of statins in human cancer suppression or patient’s prognosis, the efficacy of statitin tretment might be different among type or dose of statins, cancer type, and type of genetic alterations in tumors [66, 69]. Other inhibitors for the mevalonate pathway have been tested in clinical trials with promising results such as zoledronic acid, farnesyl transferase inhibitors (FTIs) and GGTI inhibitors. TP53 status, which is not considered in these clinical trials, might be an important variable to predict the response to mevalonate pathway inhibitors [70].

An increasing number of studies highlight the role of mutp53 proteins in the alteration of cancer cell secretome and in the modification of tumour microenvironment [25, 61]. Solid tumours take advantage of a co-evolution of neoplastic and stromal cells. Extracellular matrix (ECM) also plays a dynamic role in cancer invasion [71]. Cancer cells secrete cytokines, chemokines, proteases, growth and angiogenic factors that are able to regulate the crosstalk between stroma/cancer cells and tumour microenvironment [72]. Recent evidence have demonstrated a key role of mutp53 in the aberrant modulation of the extracellular matrix (ECM) components [60, 73, 74], in the secretion of pro-inflammatory, immunomodulatory interleukins and cytokines [60, 75, 76] and in the regulation of the crosstalk between tumour and stromal cells [19, 60, 61, 77].

An additional pathway in which mutp53 plays a role is autophagy. Autophagy has been widely recognized as a main pathway involved in both the regulation of cancer cell proliferation and in the response to several anticancer drugs. It is an intracellular degradative process through which damaged macromolecules and organelles are targeted to lysosomes via autophagic vesicles.. Originally Kroemer and colleagues described the relationship between mutp53 proteins and autophagy. Indeed, they showed that the cytoplasmic localization of mutp53-R273H and mutp53-R273L inhibited the autophagy machinery [78]. On the contrary, the mutp53 proteins preferentially localized in the nucleus, such as mutp53-P151H and mutp53-R282W, failed to counteract autophagy [78]. AMPK is a highly conserved serine/threonine protein kinase complex that, induced by metabolic stress, stimulates autophagy as tumor suppressor mechanism by phosphorylation of several downstream transcription factors, wtp53 among them, and metabolic enzymes [79]. Recently, It has been documented that both contact and conformational mutp53 proteins prevent autophagy in cancer cells through the constitutive inhibition of AMPK signaling [25]. Indeed, mutant p53 significantly counteracts the formation of autophagic vesicles and their fusion with lysosomes throughout the repression of some key autophagy-related proteins and enzymes such as BECN1 (and P-BECN1), DRAM1, ATG12, SESN1/2 and P-AMPK. This also pairs with the stimulation of mTOR signalling pathway [25]. These finding are in agreement with a number of works indicating that mutp53 proteins exert their oncogenic functions also through the stimulation of PI3K/Akt/mTOR signaling in order to inhibit autophagy and promote cancer proliferation [59, 80]. Interestingly mutant p53-driven mTOR stimulation sensitizes cancer cells to the treatment with the mTOR inhibitor everolimus. This might suggest the use mTOR inhibitors for the treatment of patients carrying mutant p53 proteins [25]. Functionally, Choudhury and collegues showed that mutp53 stability increased in cells treated with the autophagic inhibitor chloroquine, while overexpression of key autophagic genes led to mutp53 protein degradation [81]. Moreover they found that glucose restriction leads to proteasome-independent but autophagy-dependent degradation of mutp53 proteins [82]. They reported that mutp53 binds proteins belonging to the autophagic apparatus in lysosomes [82]. These metabolic changes mutp53-induced as a novel GOF to promote tumor development are a strong rationale to study glycolytic and mitochondrial oxidative phosphorylation pathways. The enhanced glucose metabolism under aerobic conditions (Warburg effect) is a common feature of many tumors to meet the high biosynthetic demand of rapidly dividing cells [83]. However increased glycolysis is observed in many types of cancer cells, but this metabolic shift can also cause increased oxidative stress and DNA damage [84]. A recent study reported that mutp53 promotes glycolysis and the Warburg effect in both cultured cells and mutant p53 R172H knock-in mice as an additional novel GOF of mutp53 [84]. Furthermore mutp53 induces the expression of glycolytic enzyme hexokinase II, which could increase glycolysis [85].

**Fig. 1:**
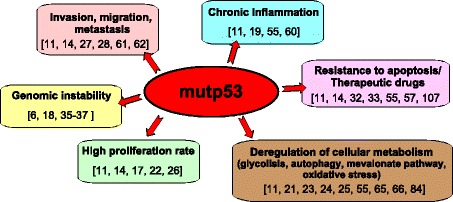
Mutant p53 involvement in pathways associated with cancer development**.** Mutant p53 regulates many cellular processes, such as proliferation, migration, invasion, survival, metabolism, chemoresistance, and tissue architecture, to promote tumor progression

Collectively, these findings mirror a pivotal role of mutp53 in mediating cancer metabolic alteration, thereby depicting a novel mechanism underlying mutant p53 gain-of-function in human cancers (Fig. 1).

### Therapies to restore wild-type p53 functions

In preclinical studies reactivation of wild-type p53 in p53-null or p53 mutant tumors is sufficient to slow down or regress the tumor progression [86–90]. This finding has stimulated the research of multiple approaches to reactivate wild-type p53 functions in tumor cells carrying mutant p53 proteins.

**Fig. 2:**
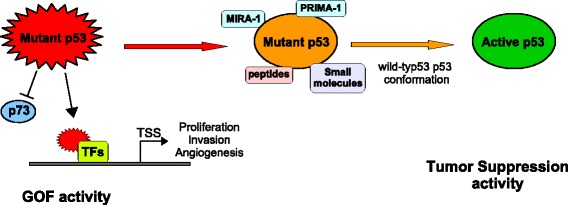
Mutant p53 is targeted by small molecules. PRIMA-1, MIRA-1, diverse kind of peptides and small compounds bind to mutantp53 and restore wild-type p53 tumor suppressor abilities. Moreover, they block mutantp53-induced inhibition of TAp73 restoring its transactivation activity

Small molecules have been developed that specifically target mutant forms of p53 restoring p53 transcriptional activity, thereby leading to cell cycle arrest or apoptosis of tumor cells (Fig. 2).

The small molecules PRIMA-1 and MIRA-1 have been identified in a cell-based screen of two thousands compounds from the National Cancer Institute (NCI) library [91, 92]. Unfortunately MIRA-1 was not further followed up due to high toxicity [92, 93].

PRIMA-1 and its analogous PRIMA-1MET (APR-246), can restore mutant p53 proteins to a wild type conformation re-establishing a wt-p53 transcriptional activity. This leads to enhanced expression of Puma, Noxa and Bax in p53 mutant cells [93, 94]. Chemically PRIMA-1 is converted intracellular to Michael acceptor methylene quinu- clidinone (MQ) that binds covalently to cysteine residues of mutant p53 protein [95]. Using computational prediction of p53 structural models, a transiently open pocket close to Cys124, Cys135 and Cys141 was identified as a potential binding site for MQ [96]. Binding of MQ leads to refolding of the mutant proteins. This refolding is highlighted by a differential interaction between APR-246 pre-treated and post-treated p53 with conformation-specific antip53 antibodies and by mass spectrometry analysis [97].

In vivo, APR-246 has shown impressive cytotoxic and apoptotic effects in murine cancer models of such as mutant p53 small cell lung carcinoma [97], multiple myeloma [98] and breast cancer [99]. APR-246 was tested in 2012 in a phase I/IIa clinical trial of 22 patients with hematologic malignancies and prostate cancer (TP53 mutation status was not a pre-selection criterion). The drug was generally well tolerated with only transient side effects such as fatigue, dizziness, headache, and confusion. Two minor responses were observed in these late stage patients, one in an acute myeloid leukemia (AML) patient carrying V173 M mutant p53 and the other one in a non-Hodgkin lymphoma patient with a TP53 splice site mutation [100]. Currently patients are enrolled in diverse clinical studies:

1. Phase Ib/II study for the treatment of either platinum resistant advanced and metastatic oesophageal or gastro-oesophageal junction cancers bearing mutant p53 proteins (NCT02999893).
2. Phase Ib/II study for the treatment of platinum sensitive recurrent high-grade serous ovarian cancers with mutated p53 with APR-246 in combination with carboplatin and pegylated liposomal Doxorubicin Hydrochloride (PLD) (NCT02098343).
3. Phase 1b/II study for the safety and efficacy of APR-246 w/azacitidine in mutant p53 myeloid neoplasms (NCT03072043).
4. Phase II study in platinum-resistant high-grade serous ovarian cancers with APR-246 in combination with PLD (NCT03268382).

These clinical trials are still enrolling patients and no data have been released yet.

Recently, by multi-omics approaches, the proteasome machinery has been identified as a common target of diverse mutant p53 missense proteins in triple-negative breast cancers (TNBC) [24]. Proteasome inhibitors such as bortezomib and carfilzomib are FDA- clinically approved drugs for the treatment of multiple myeloma [101]. Although several studies support a therapeutic potential of the proteasome inhibitors in solid tumours the described resistance mechanisms have not allowed these therapies to progress beyond clinical trials [101]. Walerych and colleagues reported that the cooperation of mutp53 with Nrf2 (NFE2L2) activates proteasome gene transcription, resulting in resistance to the proteasome inhibitor carfilzomibin in TNBC [24]. On the basis of these findings, the authors simultaneously targeted mutp53 proteins and their downstream pathway-the proteasome-in TNBC cells with APR-246, thereby overcoming resistance to pharmacological inhibition of the proteasome [24]. This treatment strategy may also overcome the limitations of therapies that target only mutp53 in solid tumours [102].

### Peptides and molecules to “correct” mutant p53 activities

Most of the gain-of-function mutant p53 proteins derive from missense mutations residing in the core domain [14]. These point mutations found in the core domain thermally destabilize p53. The core domain of wild-type p53 has a melting temperature of 44 °C and a short half-life of 9 min at 37 °C [102] while mutant p53 has an increased half-life which strongly contributes to the emergence of gain-of-function phenotypes [103, 104].

This might be one of the main interest to discover peptides and small molecules that can stabilize mutant p53 in its active biological conformation, thus restoring its DNA-binding ability and potentially rescuing wild type p53 activity (Fig. 2).

p73 and p63 proteins show a high degree of structural and functional homology to p53, in particular within the DBD (around 65%) [105]. p63 and p73 can form homo- and heterotetramers only with each other, but mutp53 can interact with both p63 and p73 different isoforms sequestering them and inhibiting their transcriptional activity [12, 105, 106]. This broad inhibition of p53 family proteins results in enhanced proliferation and resistance to chemotherapy [12, 107]. Thus, inhibition of the mutp53/p73 and of mutp53/p63 protein complexes might be a promising alternative to the mutp53 refolding approach [108].

We have engineered short synthetic peptides (10–15 residues) capable to physically disassemble the protein complex mutp53/p73 [109]. The sequence of the short peptides recapitulates that of the specific DNA binding domain of p73 that is directly involved with the interaction with mutant p53 protein. Transduction of the short interfering peptides (SIMPs-**S**hort **I**nterfering **M**utantp53 **P**eptides) in tumor cells bearing mutant p53 proteins enhanced cisplatin and adriamycin-induced apoptotic response. This occurred through the specific disassembling of the protein complex mutp53R175H/p73 by SIMP5 and of the protein complex mutp53R273H/p73 by SIMP1 that provoked the restoration of p73-mediated transcription and apoptosis [110]. SIMPs did not have any effects on both p53 null and wt-p53 expressing cells, thereby indicating that SIMPs activity is strictly connected to the presence of mutant p53 protein.

With a different approach, Del Sal’s group reported that short peptide aptamers (PA) able to bind to different p53 mutants, whereas not to wt p53, were identified and characterized [110]. PAs consist of a short variable peptide domain usually expressed in the context of a protein scaffold and they are selected from high-complexity libraries to specifically target proteins and modulate their activity [111]. The identified PAs specifically interfere with mutant p53 transcriptional functions and they have a specific ability to induce cell death in different cell lines expressing mutant p53, whereas the same PAs failed to produce any effect in cells bearing wt p53 or in p53-null cells.

Recently, Tal and coworkers approached a phage display screening to select mutp53-reactivating peptides [112]. They found that the analyzed common p53 mutants were bound and reactivated by these peptides. Moreover, they promoted selective apoptotic death of cancer cells carrying mutant p53, and decreased the growth of human cell line-derived mouse xenograft tumors representing several types of highly aggressive cancer types. Remarkably, some peptides bear similarity to the sequences of human proteins known to interact with p53 suggesting that the peptides selected can indeed interact with p53 in a functionally relevant manner [112].

The hypothetical mechanism underlying the activity of all these peptides might be accounted for the fact that proteins of a typical population of mutant p53 in the cell are in a constant dynamic equilibrium between the misfolded and properly folded conformations. The peptides by binding preferentially to mutant p53 when it transiently exhibits a wild type conformation might stabilize it and gradually shifts the population equilibrium towards the wild-type conformation.

It is estimated that 75,000 new cancer cases per year bear Y220C p53 mutation. This mutation, in the core domain of p53 is not involved in DNA binding, but it creates a cavity that destabilizes p53 protein. The small molecules PhiKan083 and PhiKan7088 bind to this cavity and therefore interact with Y220C p53. In vitro, PhiKan7088 corrected the folding of Y220C p53 restoring transactivation potential inducing p21 and Noxa expression with the consequent cell cycle arrest and apoptosis [113].

Recently, El-Deiry group has described two additional mutant p53-targeting small molecules: (a) prodigiosin that disrupts the interaction of mutant p53 with the p53 family member p73, and thereby unleashes the cytotoxic and cytostatic activities of p73 [114]; (b) NSC59984 that augments mutant p53 degradation and also unleashes p73 activity [115].

Garufi et al., found that capsaicin, the major constituent of peppers, induced mutant p53 degradation and restored wild type p53 functions such as DNA binding and transactivation of target genes [11]. Furthermore, in combination with chemotherapeutic agents, capsaicin induced cell death suggesting that capsaicin-induced p53 reactivation may improve mutp53-carrying cancer cell response to chemotherapy [116].

The role of mutant p53 protein as a target for dietary-related cancer chemopreventive compounds is a new field of research. Aggarwal and collegues have recently showed that cruciferous-vegetable-derived phenethyl isothiocyanate (PEITC) can reactivate p53 mutant under in vitro and in vivo conditions, revealing a new mechanism of action for a dietary-related compound [117]. PEITC exhibited growth-inhibitory activity in cells expressing p53 mutants with preferential activity toward mutant p53R175H. Mechanistic studies revealed that PEITC induced apoptosis in a p53R175H mutant-dependent manner by restoring wtp53 conformation and transactivation functions. Interestingly, the growth inhibitory effects of PEITC depended on the redox state of the cell. PEITC treatments render the mutant p53R175H sensitive to degradation by the proteasome in a concentration-dependent manner. Furthermore, the dietary supplementation of PEITC was able to reactivate WT p53 activity in vivo as well, inhibiting tumor growth in xenograft mouse model [117]. These findings provide the first example of mutant p53 reactivation by a dietary compound and have important implications for cancer prevention and therapy.

Bringing small peptides and molecules into the clinic remains challenging, mainly owing to the need to deliver the peptides efficiently into the tumor cells. However, their greater specificity bears the hope for minimal non-specific toxicity, rendering such approach highly promising in the long run.

### Genomic instability to target mutant p53 in human cancers

Genomic instability may arise from different pathways as centrosome amplification, epigenetic modifications, telomere dysfunction and DNA damage from endogenous and exogenous sources. In the presence of genomic instability there is an increase in the rate of DNA alterations compared to normal cells [118]. Oncogenic mutantp53 proteins promote both chromosomal (CIN) and amplification (AIN) instabilities [35–37]. Notably, expression of mutp53R172H (corresponding to human R175H) in p53-null primary mouse mammary epithelial cells and developing mouse mammary tumours resulted in aberrant centrosome amplification, multipolar mitoses and increased numbers of chromosomes [38, 39].

In vitro data also suggest that mutp53 can facilitate structural chromosomal abnormalities by interacting with and inhibiting the genome caretaker proteins of DNA repair. The fidelity of DNA double strand break (DSB) repair plays a central role in preventing translocations. In response to DNA double strand break signals, cells have at their disposal two distinct repair mechanisms: homologous recombination (HR) and nonhomologous end joining (NHEJ) [119]. MRE11 is a DNA binding protein involved in both HR and NHEJ [120]. Some mutant p53 proteins have been shown to bind MRE11 sequestering it from the DNA site of double strand breaks, thus increasing the amount of spontaneous chromosome, chromatid breaks and translocations, which happens in MRE11 null cells [121]. By this way mutant p53 abolishes the phosphorylation signaling which culminates with activation of ATM, the principal double strand break sensor in cells to activate HR, resulting in bypassing of the G2/M DNA damage checkpoint and a decrease of genomic stability [122].

Recently, we have documented that transcriptional activity of GOF mutant p53 proteins plays a role in the inefficient activation of DNA repair mechanism and consequent DNA damage accumulation in proliferating tumour cells [18]. In search for co-factors sharing mutant p53-induced transcriptomic alterations in cancer cells, we identified the transcriptional inhibitor E2F4 as a new partner of mutant p53 proteins in diverse types of tumor cells. E2F4 plays an important role in the suppression of proliferation-associated genes and recent evidences report that E2F4 may play an oncogenic rather than a tumor suppressor role in cancer cells [123]. Mutant p53/E2F4 oncogenic complex is able to bind *rad17* and *brca1* gene promoters inhibiting their expression. Both BRCA1 and RAD17 proteins are key signal transducers during checkpoint activation in the response to DNA DSBs [124]. Furthermore the concomitant recruitment of mutant p53 and E2F4 proteins onto *rad17* and *brca1* gene promoters provided a global increase of histone H3 methylation and a decrease of histone H4 acetylation [18]. This might contribute to chromatin transcriptional inactive status of *rad17* and *brca1* promoter regions.

From a clinical point of view, it was considered a cohort of tumors from head and neck squamous cell carcinoma (HNSCC) patients where *TP53* status was assessed by direct sequencing of exons 2 through 11 [125]. HNSCC is characterized by a high grade of genomic instability and a *TP53* mutation incidence of nearly 62% [126]. In these patients *rad17* and *brca1* were expressed at lower level in tumors when compared to non-tumoral matched samples. This was significantly striking in the group of patients carrying mutant p53 proteins independently from other clinic-pathological parameters. Unlike those with mutant p53, wild type p53 tumors did not show any significant difference for *rad17* expression between tumor and normal groups. Interestingly, *brca1* transcript was upregulated in wild type p53 tumors [18]. Collectively, these findings strongly support the hypothesis of an active repression of *rad17* and *brca1* gene expression by mutant p53 proteins, leading to a continuous DNA DSBs accumulation with a permanent increase in genomic instability.

**Fig. 3:**
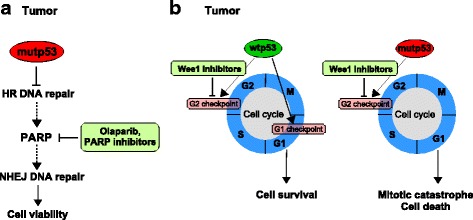
Selective killing of mutant p53 tumor cells through cell-cycle checkpoint abrogation. (**a**) Endogenous single strand breaks (SSBs) are repaired by SSB repair pathways that involve PARP1. If base excision repair (BER) is impaired, through the inhibition of PARP (for example by olaparib), single strand breaks become double strand breaks. In patients with HR defects, such as a BRCA mutation carrier, this damage causes the cancer cell death since PARP inhibitors induce aberrant activation of NHEJ. (**b**) Compared to wtp53 cells with an intact G1 checkpoint, mutp53 cancer cells lacking the p53-dependent G1 checkpoint may depend more on the G2 checkpoint to survive after DNA damage. The inhibition of Wee1 activity could lead to mitotic catastrophe and cell death

Poly (ADP-ribose) polymerase 1 and 2 (PARP) are nuclear proteins that are activated by DSBs [127]. PARP1 has the function to protect DNA breaks and chromatin structure and to recruit DNA repair and checkpoint proteins to the sites of damage [128]. Interestingly, inhibition of PARP1 was found to be synthetically lethal for cells with defects in homologous recombination HR [129], a DSBs repair mechanism, and was particularly effective in tumor cells that lack functional BRCA1 or BRCA2 [129, 130]. DSBs repair depends on HR and NHEJ. To explain the synthetic lethality effect of PARP-inhibitors it has to say that PARP1 is involved in NHEJ [131], cancer cells with deficiency in BRCA or HR will thus require PARP1-dependent NHEJ for DSBs repair, and become more vulnerable to apoptosis when PARP1 is inhibited [129] (Fig. 3A). If the DNA repair function of HR is intact, the inhibition of PARP1 alone may not necessarily induce cell death. PARP1 inhibitors such as olaparib and niraparib have been tested in clinical trials in breast and ovarian cancers [132, 133] (Fig. 3A). Recently, olaparib was approved by the FDA as a monotherapy for the treatment of patients with deleterious or suspected deleterious germline BRCA mutation in advanced ovarian cancer who have been treated diverse prior lines of chemotherapy [134]. PARP inhibition is also promising for triple negative breast cancer (TNBC), which is an aggressive breast cancer characterized by high levels of replicative stress due to c-MYC amplification, EGFR activation and TP53 mutations (p53 gene was found to be mutated in approximately 80% of basal/TNBC) [135, 136]. Importantly, up to 20% of TNBC patients harbour germline BRCA mutations. Additionally, patients with sporadic TNBC without BRCA mutation show BRCA1 mutation-like tumor conditions (“BRCAness”) in which BRCA is inactivated by other mechanisms such as promoter methylation or gene expression inhibition [18, 137, 138]. All these evidences strongly suggest that impaired HR from either BRCA mutation or BRCAness, PARP inhibition is believed to be a rational approach for this subtype of breast cancer. To confirm this, olaparib in phase II studies did not result in significant positive responses in non-BRCA-associated TNBC [139]. Combination strategies with olaparib and chemotherapeutic agents have shown to be effective in ERCC1 or PTEN-deficient lung cancer cells, showing that other deficiencies in DNA-repair pathways frequently occurring in NSCLC might have an impact on the response to olaparib treatment, and consequently also on this combination strategy [140, 141]. Therefore, further studies on the genetic profile of patient tumors might lead to the identification of other predictive markers besides the TP53 status.

The disruption of the nuclear architecture of telomeres might be another novel pathway through which TP53 mutants induce genomic instability in cancer cells. A pancreatic cancer model expressing endogenous expression of Trp53(R172H) and Kras(G12D) demonstrated a high degree of genomic instability manifested by nonreciprocal translocations [142]. Furthermore, the authors showed that significant portions of telomeric sequences at the fusion points of translocations while the telomere sequences at the ends of chromatids were conserved, a phenotype observed in cells which lost the function of telomere capping proteins [143]. Previous reports showed the efficacy of the telomerase template antagonist, GRN163L, as a potential anticancer agent in metastatic breast cancer MDA-MB-231 cells expressing mutp53R280K [144, 145]. It was observed that GRN163L induced a rapid change in cellular architecture, leading to the hypothesis that GRN163L can augment the effects of the microtubule stabilizer paclitaxel in reducing the cell growth of breast cancer cells [144]. The combined treatment of GRN163L and paclitaxel resulted in a significant synergistic combination index (CI), in reducing the cellular proliferation and invasive potential of MDA-MB-231 breast cancer cells. Interestingly, a randomized Phase II study of imetelstat (grn163l) in combination with paclitaxel (with or without bevacizumab) in patients with locally recurrent or metastatic breast cancer has been completed and the results are expected (NCT01256762).

### Targeting WEE1 protein kinase to disarm mutant p53

WEE1 is a tyrosine kinase with a pivotal role at the G2-M cell cycle checkpoint that prevents entry into mitosis in response to cellular DNA damage [124]. The progression from G2 into M phase is controlled by the Cdk1/Cyclin B complex that is activated by dephosphorylation of tyrosine 15 (Tyr15) on Cdk1 by the Cdc25c phosphatase [124]. Before mitosis Cdk1 is maintained in an inactive state by WEE1 through phosphorylation of CDK1 at tyrosine 15 [124, 146]. WEE1 is highly expressed in several cancer types, including hepatocellular carcinoma [147], cervical cancers [148], lung cancers [149], squamous cell carcinoma [148, 150], colorectal cancers [151], gastric cancers [152], leukemia [153, 154], melanoma [155], and ovarian cancers [156]. High expression of WEE1 has been reported in some cancers in response to elevated replication stress, and has been associated with tumor progression and poor outcome [148, 155, 156] (Fig. 2B). Loss of WEE1 activity sensitizes p53 inactive cells to DNA damaging agents and radiosensitization [157–160]*. Recently Moser and colleagues performed RNAi kinome viability screens in HNSCC to identify novel therapeutic drug targeting mutant p53 protein* [150]*.* Kinase targets were selected on the basis of impaired viability and increased apoptosis following kinase knocking-down. Putative survival kinases included signaling proteins within the focal adhesion and integrin (CAMK2B, FYN, ILK, EPHA3, EIF2AK4, TRIB2), PI3K signaling (PIK4CB, PIK3CB, PIP5K1B, TRIB2, FGFR3, ALK), SRC signaling (FYN, TXK, CAM2KB), and G2/M cell cycle regulation (WEE1, NEK4, TTK, AURKA, CHK1). WEE1 was implicated as a critical survival kinase for TP53 mutant HNSCC cells. Treatment with WEE1 inhibitor MK-1775 caused unscheduled mitotic entry and apoptotic death selectively in mutp53 versus wild-type p53 cell lines. It also increased cisplatin-induced killing in a mutp53 orthotopic xenograft model [150]*. I*n patients with HNSCC undergoing curative-intent surgery and heterogeneous adjuvant therapy, TP53 mutations were associated with reduced survival, independent of pathologic nodal stage or primary tumor site [161]. TP53 mutations were validated as a prognostic biomarker in a cohort of patients treated homogeneously with primary surgery and postoperative radiotherapy [162]. Further our group analyzed *TP53* status by direct sequencing of exons 2 through 11 of a prospective series of 121 HNSCC samples and assessed its association with outcome in 109 followed-up patients [125]. A *TP53* mutation was present in 58% of the tumors and *TP53* mutations were significantly associated with a shorter recurrence-free survival [125]. In an orthotopic murine model evaluating 48 validated HNSCC cell lines, TP53 mutations correlated with higher growth rate, cervical nodal metastases, and decreased survival, suggesting a biologic basis for inferior prognosis [163]. Cells carrying wtp53 protein arrest at the G1 checkpoint of the cell cycle to repair damaged DNA, before DNA replication (Fig. 3B). Cells with defective p53 pathway as for those carrying mutant p53 proteins rely mainly on DNA repair at the G2 checkpoint [164]. Indeed several inhibitors of the G2 checkpoint sensitize mostly mutp53 tumor cells to DNA-damaging agents [165].

These evidences support the mitotic lethality rationale that cancers deficient in functional p53, a key component of the G1-S checkpoint, are more reliant on the G2-M checkpoint to repair DNA damage, and that abrogation of the G2-M checkpoint by WEE1 inhibition sensitizes p53-deficient cells to DNA-damaging agents [166] *(*Fig. *3*B*)*. In support of this, it has been previously documented that Cdk1/Cyclin B protein complex activity is higher in mutant p53 cells and is reduced by mutant p53 depletion [17, 22]. This suggests that abrogation of the G2-M checkpoint will selectively impact on tumor cells, with only limited effect on normal cells that have functional G1 and G2-M checkpoints. Therefore, inhibition of WEE1 kinase activity and removal of the G2-M checkpoint is an attractive strategy to drive cancer cells to enter into unscheduled mitosis and ultimately undergo cell death via mitotic catastrophe [167] (Fig. 3B). This principle was first demonstrated in mutp53 colorectal carcinoma, in which the preclinical WEE1 kinase inhibitor, PD0166285, potentiated radiation-induced killing by abrogating G2-M arrest and forcing premature mitotic entry [168].

A screening program that focused on the identification of WEE1 inhibitors from a small chemical compound library led to the discovery of the potent WEE1 inhibitor AZD1775 (also known as MK1775) [169]. There are currently 43 clinical studies listed in ClinicalTrials.gov for AZD1775 (MK1775), covering a wide range of cancer types including solid tumors, leukemia, childhood and adult brain tumors. In most of these studies AZD1775 is used in combination with carboplatin, cisplatin, docetaxel, 5-fluorouracil, gemcitabine, irinotecan, paclitaxel, pemetrexed, temozolomide, topotecan, or irradiation (clinicaltrials.gov). Of great interest those studies enrolling patients with TP53 mutations including: (a) the Phase II pharmacological study with MK-1775 combined with carboplatin in patients with p53 mutated epithelial ovarian cancer (NCT01164995); (b) the phase II study evaluating MK-1775 in combination with paclitaxel and carboplatin versus paclitaxel and carboplatin alone in adult patients with platinum sensitive p53 mutant ovarian cancer (NCT01357161); (c) the phase II single-arm study of AZD1775 monotherapy in relapsed small cell lung cancer patients with myc family amplification or CDKN2A mutation combined with TP53 mutation (NCT02688907); (d) the randomized phase II trial of cisplatin with or without WEE1 kinase inhibitor AZD1775 (MK-1775) for first-line treatment of recurrent or metastatic squamous cell cancer of the head and neck (NCT02196168). All these studies are actively enrolling patients and the related data will be expected.

## Conclusions

Because of its high mutation rate and its critical role in driving cancer formation/progression, the therapeutic targeting of mutant p53 is of absolute priority. Compounds reactivating mutant p53 to wild-type p53 tumor suppressor activities are actually available. Size, polarity, solubility, pharmacokinetic and pharmacodynamic properties of these compounds can be improved through medical chemistry approaches and drug-protein interaction studies. There are still many unresolved questions surrounding the role of mutant p53 in cancer. As the knowledge on the molecular mechanisms underlying gain of function mutant p53 protein progresses its therapeutic targeting will be much more precise and hopefully much more successful.
